# Cross-Protection in PRRSV: Mechanisms, Limitations, and Implications for Vaccine Design

**DOI:** 10.3390/pathogens15040345

**Published:** 2026-03-24

**Authors:** Sergei A. Raev, Limeng Cai, Nina Muro, Rachel Madera, Lihua Wang, Jishu Shi

**Affiliations:** 1 Center on Biologics Development and Evaluation, College of Veterinary Medicine, Kansas State University, Manhattan, KS 66506, USA; 2 Department of Anatomy and Physiology, College of Veterinary Medicine, Kansas State University, Manhattan, KS 66506, USA

**Keywords:** PRRSV-1, PRRSV-2, cross-protection, heterologous immunity, neutralizing antibodies, T-cell mediated

## Abstract

Porcine reproductive and respiratory syndrome (PRRS) remains one of the most economically devastating diseases in global swine production. The causative agent, PRRS virus (PRRSV), comprises two genetically distinct species—PRRSV-1 and PRRSV-2—that differ substantially in antigenic composition and immune recognition. Despite widespread use of modified live vaccines (MLVs), protection against heterologous and cross-species strains remains inconsistent and difficult to predict. This review synthesizes current knowledge of homologous, heterologous, and cross-species protection, with emphasis on humoral and cellular immune responses and the viral determinants that constrain breadth of immunity. Neutralizing antibodies can confer near-sterilizing homologous protection under controlled conditions; however, their delayed induction and narrow specificity limit efficacy against heterologous strains. T-cell-mediated responses are generally broader but remain highly strain- and context-dependent. Structural features of PRRSV envelope glycoproteins, including glycan shielding and immunodominant decoy epitopes, further restrict antibody-mediated cross-protection while providing targets for rational vaccine design. We also examine potential drawbacks of preexisting immunity, including antigenic mismatch and non-neutralizing antibody-dominated responses that may contribute to suboptimal outcomes following heterologous exposure. Collectively, these findings highlight the multifactorial nature of PRRSV protection and the need for next-generation vaccines capable of inducing broader and more durable immunity.

## 1. Introduction

Porcine reproductive and respiratory syndrome (PRRS) is a leading cause of economic loss in the global swine industry, resulting in reproductive failure in sows and respiratory disease in growing pigs. The disease is caused by two distinct viral species, PRRSV-1 and PRRSV-2, which exhibit rapid evolutionary dynamics driven by high mutation rates, extensive intra-host diversity, and frequent recombination [1]. These species share only approximately 50–70% nucleotide identity across their genomes, reflecting profound genetic divergence that has important implications for immunity, vaccine performance, and cross-protection.

Given the limited heterologous protection observed even among closely related PRRSV strains, cross-species protection has traditionally been considered unlikely [2]. Nevertheless, epidemiological surveillance indicates a marked geographic separation in PRRSV species dominance, with PRRSV-1 predominating in much of Europe and PRRSV-2 remaining dominant in North America and large parts of Asia, reflecting both historical introduction events and ongoing transmission dynamics [3,4]. Notably, despite multiple documented introductions of both species into non-endemic regions, sustained dominance by a single PRRSV species is largely maintained. This pattern may, in part, reflect the influence of cross-species immune interactions, although alternative explanations—such as differences in viral fitness, transmission efficiency, vaccine usage, and population immunity—are also likely to contribute.

Both experimental and field observations indicate that protection against PRRSV exists along a continuum rather than as a binary outcome. Protective immunity to PRRSV is multifactorial and remains difficult to predict, particularly in the context of extensive viral genetic diversity and widespread vaccine use. In this review, we analyze available experimental and field literature through this conceptual framework to illustrate how reported protective outcomes span a continuum of virological and clinical effects. Homologous protection refers to immunity directed against the same or very closely related PRRSV strains and is typically associated with marked reductions in viremia and clinical disease. Heterologous protection denotes protection against genetically distinct strains within the same PRRSV species (PRRSV-1 or PRRSV-2), whereas cross-species protection describes immunity across the two PRRSV species.

**Figure 1:**
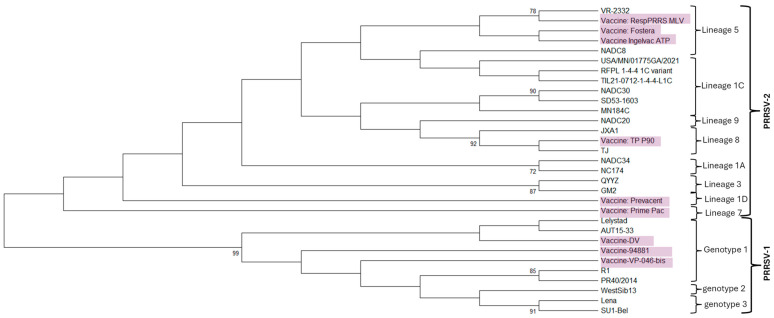
Phylogenetic relationships among PRRSV strains discussed in this review. A Neighbor-Joining phylogenetic tree was constructed based on nucleotide sequences of the ORF5 gene using Mega12 software. Bootstrap support values (%) from 1000 replicates are shown at branch nodes. The tree includes representative PRRSV-2 and PRRSV-1 strains selected based on their epidemiological relevance, lineage or genotype classification, and use in commercial vaccines or experimental studies referenced in this review. Major PRRSV-2 lineages and PRRSV-1 genotypes are indicated on the right. Commercial vaccine strains are highlighted. PRRSV-2 vaccine strains cluster within a relatively narrow subset of lineages, primarily associated with classical lineage 5 and related genetic backgrounds, whereas contemporary field strains—including L1C variants—form distinct phylogenetic clusters. Similarly, PRRSV-1 vaccine strains group within limited genotype clusters and show genetic separation from circulating field strains, indicating constrained vaccine diversity across both PRRSV species. This phylogenetic framework provides context for the variability in homologous and heterologous protection discussed in the text and underscores the potential limitations of the restricted genetic diversity of existing vaccine strains in addressing emerging PRRSV variants.

The phylogenetic relationships of representative PRRSV strains referenced in this review are shown in Figure 1, and the list of ORF5 sequences used for the phylogenetic analysis is provided in Table 1.

In the second part of this review, we examine virus- and host-related factors that contribute to this continuum of protection. Multiple components of humoral and cellular immunity have been proposed as correlates of protection [5,6,7]. Passive transfer of immunoglobulins with neutralizing antibodies can confer sterilizing homologous protection under controlled experimental conditions; that said, PRRSV infection is typically evidenced by delayed induction and strain-restricted neutralizing breadth, driven at least in part by PRRSV-specific immune evasion and virulence mechanisms, which limit the contribution of neutralizing antibodies to heterologous protection [8,9]. In contrast, T-cell-mediated responses tend to be broader but remain strongly influenced by viral strain and immunological context [10,11]. Finally, we analyze factors associated with broadened cross-protection and discuss their implications for evaluation of current and future vaccine strategies.

## 2. Cross-Species Protection Between PRRSV-1 and PRRSV-2: An Underappreciated Phenomenon

Epidemiological surveillance has shown that PRRSV-1 predominates in many parts of Europe, particularly Western and Eastern Europe, whereas PRRSV-2 is the dominant species in North America and much of Asia, reflecting both historical introduction patterns and ongoing transmission dynamics. Given the limited heterologous protection observed even among closely related PRRSV strains, the absence of meaningful cross-species protection has long been considered the default expectation [12,13]. Despite this expectation—and despite frequent co-circulation of PRRSV-1 and PRRSV-2 in multiple regions—a marked predominance of one species over the other is often maintained.

For instance, PRRSV-1 was originally the only circulating species in European countries, and even after the introduction of PRRSV-2 through modified live virus (MLV) vaccination [14] in 1996 and other independent introductions, including non-vaccine-related strains [15,16], PRRSV-2 remains sporadic in the European Union [3,17]. Similarly, PRRSV-2 has been reported only sporadically in Russia [18], including reports of highly pathogenic strains [19]. Conversely, PRRSV-1 has been detected in the United States, Canada, China, Korea, and Thailand—regions where PRRSV-2 predominates—but its overall prevalence remains low [20,21,22]. In the United States, for instance, PRRSV-1 accounts for no more than ~2% of reported PRRSV isolates based on recent surveillance data [23]. Notably, despite the low prevalence of PRRSV-1 in this setting, sera collected from sows have been shown to neutralize a range of PRRSV-2 strains as well as PRRSV-1 strains, an observation consistent with—but not proof of—partial cross-species humoral immunity [24].

Experimental studies generally suggest that cross-species protection between PRRSV-1 and PRRSV-2 is limited, although the available data are relatively sparse and heterogeneous. Several early investigations reported that PRRSV-2-based vaccines failed to confer protection against PRRSV-1 challenge in pigs, sows, or boars, with minimal effects on viremia or virus shedding [25,26]. However, other studies have described partial protection following PRRSV-2 MLV vaccination, including reductions in clinical disease severity or viral replication after PRRSV-1 challenge, although these effects were variable and context-dependent [27,28,29].

Consistent with these in vivo observations, in vitro experiments have suggested that prior PRRSV-2 infection may suppress subsequent PRRSV-1 replication, whereas the reverse effect appears less pronounced [30]. While these findings support the possibility of asymmetric cross-species interference, their biological relevance under field conditions remains uncertain. Nevertheless, this asymmetry may partially contribute to the limited establishment of PRRSV-1 in PRRSV-2–endemic regions such as the United States, although this remains speculative and likely multifactorial.

Together, these observations indicate that historical introduction alone may not fully explain the sustained predominance of a single PRRSV species in different regions. Partial cross-species immune effects may contribute to this pattern; however, alternative factors such as differences in viral fitness, transmission dynamics, and surveillance intensity are also likely to play major roles [31,32,33,34].

In contrast, evidence for cross-species protection conferred by PRRSV-1 MLV vaccines against PRRSV-2 is more limited and less consistent. Most studies report little to no protection following PRRSV-2 challenge, particularly with respect to virological outcomes, although partial clinical effects have occasionally been observed [35,36]. Dual-challenge experiments suggest that any cross-species effects may be asymmetric and highly context-dependent, with some indication that PRRSV-2 MLV priming followed by PRRSV-1 challenge may yield more measurable effects than the reverse sequence [37]; however, this conclusion is based on a limited number of experimental studies.

Collectively, these findings suggest that cross-species protection between PRRSV-1 and PRRSV-2 is generally limited and incomplete, with potential asymmetry that remains insufficiently characterized. As summarized in Table 2, cross-species immunity appears to depend on vaccination order, host status, and experimental conditions, but current evidence is insufficient to draw definitive conclusions regarding its magnitude or practical relevance. Data remain sparse and inconsistent—particularly for PRRSV-1-based vaccines—highlighting the need for more systematic and standardized studies to clarify the mechanisms and significance of cross-species protection in PRRSV control strategies.

## 3. European PRRSV(PRRSV-1): Apparent Vaccine Efficacy and Hidden Gaps

PRRSV-1 exhibits substantial genetic diversity across Europe and is classified into at least three major subtypes: PRRSV-1.1, PRRSV-1.2, and PRRSV-1.3. PRRSV-1.1 is the most prevalent and geographically widespread subtype and is generally associated with mild clinical disease [39]. PRRSV-1.2 and PRRSV-1.3 are comparatively rare and geographically restricted but are more often associated with severe clinical outcomes [40,41]. Nucleotide sequence identity among these subtypes ranges from approximately 78% to 82%, reflecting substantial genetic and antigenic divergence within PRRSV-1 [4,16].

Overall, PRRSV-1 strains are considered less pathogenic than PRRSV-2 strains. In particular, PRRSV-1.1 (Lelystad-like) viruses typically cause minimal or no respiratory disease in piglets [42,43,44]. Nevertheless, infection of pregnant sows with Lelystad or closely related strains can result in reproductive disorders, including stillbirths, mummified fetuses, and weak piglets [45,46], although some studies report limited reproductive impact [47].

Consistent with their relatively low pathogenicity, vaccination with Lelystad-based MLVs generally protects against disease following homologous challenge with Lelystad-like strains [48,49,50]. An autogenous inactivated vaccine was able to reduce viremia after a homologous PRRSV-1.1 challenge but failed to protect against heterologous PRRSV-1.1 strains, whereas PRRSV-1 MLVs provided protection in both settings [51]. Limited reproductive studies indicate that PRRSV-1 MLVs can improve reproductive performance [46,52], whereas inactivated vaccines show only modest efficacy [53].

Data on heterologous protection (Table 3) are sparse. Available studies suggest that commercial PRRSV-1 vaccines may confer partial protection against respiratory disease when challenged with highly pathogenic strains such as AUT15-33, PR40, Lena, or field PRRSV-1.1 isolates [54,55]; however, protection is incomplete, and effects on reproductive performance have not been evaluated. Additionally, protection afforded by PRRSV-1.1-based vaccines against more recent European isolates, including Spanish R1 (PRRSV-1.1), BOR59, WestSib13 (PRRSV-1.2), and SU1-Bel (PRRSV-1.3), remains untested [56,57].

In contrast to subtype 1, PRRSV-1.2 and PRRSV-1.3 strains are generally more pathogenic, with some classified as highly pathogenic [40,41]. No studies have directly evaluated homologous or heterologous protection against PRRSV-1.2 strains, likely reflecting their low prevalence, restriction to Eastern Europe, and limited sequence availability in public databases [4]. PRRSV-1.3, particularly the highly pathogenic Lena strain, has been widely used as a challenge virus to assess PRRSV-1.1-based vaccines. These studies consistently demonstrate partial clinical protection, including reduced fever and viral shedding, but limited virological protection, underscoring the challenges of heterologous immunity between genetically divergent PRRSV-1 subtypes [58,59].

Comparable to PRRSV-1.2, homologous protection data for PRRSV-1.3 remain scarce due to its restricted distribution. One study showed that reinfection with Lena 46 days after primary infection did not induce clinical disease or detectable viremia and was associated with development of homologous neutralizing antibodies [60], suggesting strong homologous protection potential. However, Lena-based inactivated vaccines failed to protect pigs from severe respiratory disease despite inducing neutralizing antibodies [61].

Taken together, most data on PRRSV-1 homologous protection are derived from studies using mildly pathogenic Lelystad-like strains, which likely overestimate vaccine efficacy due to the low virulence of the challenge viruses. In contrast, protection against more pathogenic PRRSV-1 subtypes, including subtype 2 (e.g., WestSib13) and subtype 3 (Lena), remains poorly defined. These gaps hinder accurate assessment of both homologous and heterologous protection against clinically relevant PRRSV-1 strains and highlight the need for targeted studies addressing vaccine performance against highly virulent PRRSV-1 subtypes.

**Table 3:** Major PRRSV-1 lineages, representative strains, and reported vaccine protection.

PRRSV-1 Lineage/Subtype	Representative Strain(s)	Geographic Distribution	Vaccine Anchor(s)	Homologous Protection	Heterologous Protection	Key Evidence Gaps	Key References
Subtype 1 (PRRSV-1-1)	Lelystad	Europe (widespread)	Lelystad-based MLVs	High (clinical & virological)	Partial (respiratory endpoints only)	Estimates confounded by low-virulence challenge strains; reproductive protection against heterologous strains largely untested	[46,48,49,50,52]
Subtype 1 (recent variants)	AUT15-33, PR40	Europe (regional)	Subtype 1-based MLVs	Limited data	Partial (clinical only)	No systematic reproductive challenge data; strain-specific variability poorly defined	[54,55,58,62,63]
Subtype 2 (PRRSV-1-2)	WestSib13	Eastern Europe	None	Not evaluated	Not evaluated	Absence of homologous and heterologous protection studies; very limited sequence availability	[41]
Subtype 3 (PRRSV-1-3)	Lena, SU1-Bel	Eastern Europe (experimental model)	None (commercial)	High after reinfection	Partial with subtype 1 MLVs (clinical only)	No Lena-based MLV; inactivated vaccines ineffective despite NAb induction	[40,57,58,60,61,62,63,64]
Autogenous PRRSV-1 vaccines	Farm-specific isolates	Europe	Inactivated autogenous vaccines	Variable (viremia reduction)	Limited	Protection is strain- and farm-specific; minimal cross-strain applicability	[65,66,67,68]

## 4. North American PRRSV (PRRSV-2): High Diversity and Constrained Cross-Protection

PRRSV-2 is characterized by extensive genetic diversity and rapid lineage turnover, which together limit the durability and predictability of vaccine-mediated protection. Although multiple PRRSV-2 lineages circulate globally [3,69], experimental evaluation of vaccine efficacy has historically relied on a small number of older or regionally restricted strains. As summarized in Table 4, commercially available MLVs are largely anchored to lineage 5 (VR-2332–like) or lineage 8 (HP-PRRSV–like) viruses, with only limited additional representation from an L1D-based vaccine, highlighting the relatively narrow genetic diversity of current vaccine platforms.

PRRSV strains within Lineage 5, particularly sublineage 5.1 (L5A) [3], have been detected in the U.S., China, Korea, and some European countries [70]. Sublineages 5.1 (L5A) and 5.2 (L5B) comprise 3.5% and 5.1% of Lineage 5 isolates, respectively (101). The attenuated VR-2332 strain (sublineage 5.1/L5A), used in commercial MLV vaccines (Ingelvac PRRS MLV), provides near-complete protection against homologous challenge, reducing viremia, lung lesions, and clinical signs [71]. Similarly, the NADC-8 strain (sublineage 5.2/L5B) confers full protection against homologous challenge in gilts [47]. Although VR-2332-based vaccines are highly effective against homologous strains, data on their efficacy against other Lineage 5 isolates remain limited.

A key feature of PRRSV-2 Lineage 1 is its high genetic diversity (~10.5% nucleotide variation) [70], with new sublineages emerging every 1–4 years, complicating control efforts as they differ at immunologically relevant amino acid sites [72]. In the United States, Lineage 1 has remained the most prevalent and genetically diverse PRRSV-2 lineage for many years [23]. An isolate of the L1C.5 sublineage—accounting for ~39% of isolates in 2024—caused severe disease comparable to that induced by highly pathogenic lineage 8 PRRSV, highlighting the urgent need for effective vaccines against predominant L1C.5 strains [73].

Currently, there is only one commercial vaccine that targets Lineage 1 isolates. The Prevacent PRRS vaccine (Elanco), based on an L1D.2 strain [74], has been evaluated against L1A (NC174), L5 (VR-2332), L1C (NADC30) and L8 (NADC20) which share 89.6%, 87.1%, 89.6% and 87.4% ORF5 nucleotide identity, respectively [75]. This study demonstrated only partial protection of the L1D.2-based vaccine. Notably, after challenge with the closely related NA174 strain, vaccinated animals exhibited significantly higher rectal temperatures at 7 dpi [75]. In contrast, viremia and viral shedding were significantly reduced in vaccinated pigs following challenge with NADC30, NADC20, and VR2332, but not with NA174 [75]. These findings were also reflected in viral loads in bronchoalveolar lavage fluid, which were reduced in vaccinated groups after challenge with all strains except NA174, as well as in gross pathology, where vaccinated animals exhibited the highest levels of microscopic lung lesions after challenge with NA174. Finally, the L1D.2-based vaccine was able to induce a measurable neutralizing antibody (NAb) response against NADC30, VR2332, and NADC20 following challenge with the respective strains, whereas no NAb titers were detected in the NA174 group [75]. Thus, despite comparable PRRSV-specific systemic antibody responses in the NA174 group, the lack of neutralizing antibodies corresponded with poorer clinical and pathological outcomes. Although the efficacy of the L1D.2-based vaccine against L1C.5 strains has yet to be evaluated, its reduced effectiveness against the ORF5 closely related NA174 strain is concerning, underscoring the need for broader cross-protective vaccine strategies.

Lineage 8 comprises multiple highly pathogenic PRRSV (HP-PRRSV) sublineages (1–9) [76]. Several commercial vaccines derived from HP-PRRSV strains provide strong homologous protection against their parental viruses [77,78], although detailed clinical protection data are lacking for some formulations, such as the lineage 8.9 Boehringer/Ingelvac ATP (JA142/ATP) vaccine [79]. Data on heterologous protection are more limited but generally encouraging. For example, a JXA1-based (sublineage 8.7) modified live vaccine was highly effective against NADC-20 (L2.5) [80] and partially protective against NADC-30 (L1.8) [81], although another study reported no protection against a NADC-30-like strain [82]. Similarly, another HP-PRRSV-based vaccine (strain TJM-F92) conferred homologous protection against TJ-F3 and TP (sublineage 8.7) and heterologous protection against NADC-30-like strains (L1.8) [83,84]. Collectively, these findings support the notion that HP-PRRSV-derived vaccines can induce relatively broader cross-lineage protection, as discussed in the “Viral Strain Biology, Pathogenicity, and Immune Induction” section.

PRRSV Lineage 3, predominantly detected in Asian countries [85], is associated with relatively high pathogenicity [86,87]. In Taiwan, more than 75% of isolates belong to Lineage 3, although Lineage 1 is gradually increasing in prevalence [85]. Lineage 3 strains, which are closely related to Chinese HP-PRRSV [88], exhibit resistance to neutralization by hyperimmune sera raised against widely used vaccines such as HUN4-F112 and JK-100 [89]. Consistent with this, commercial L5- and L8-based vaccines generally provide limited protection against Lineage 3 viruses [88], although partial cross-protection has been reported for selected L8-based vaccines [90] and for the only commercially available L7-based vaccine [91]. To date, no vaccines based directly on lineage 3 strains are available, underscoring an urgent need for vaccine strategies that provide robust protection against lineage 3 viruses in regions where they predominate.

Several additional minor lineages, including L2–L4, L6–L7, and L9, are also recognized [3,92]. Although an L7-based modified live vaccine has recently become available, systematic data on homologous or heterologous protection for these lineages remain extremely limited [91].

**Table 4:** Major PRRSV-2 lineages, representative strains, and reported vaccine protection.

PRRSV-2 Lineage	Representative Strain(s)	Geographic Distribution	Vaccine Anchor(s)	Homologous Protection	Heterologous Protection	Key Evidence Gaps	Key References
Lineage 5 (L5)	VR-2332, NADC-8	North America; global dissemination	VR-2332-based MLVs (e.g., Ingelvac PRRS MLV)	High (clinical & virological)	Variable across lineages	Little data against non-L5 historical isolates; limited relevance to current dominant strains	[47,93]
Lineage 1 (overall)	NADC-20, NADC-30, NA174	North America, Asia	L1D-based MLV (Prevacent PRRS)	Not evaluated	Partial, strain-dependent	Dominant modern lineage lacks homologous challenge studies	[23,72,75]
Lineage 1C.5	1-4-4 L1C.5	North America (dominant since 2019)	None	Not evaluated	Not evaluated	No homologous or controlled heterologous vaccine data despite high prevalence and virulence	[23,73]
Lineage 8 (HP-PRRSV)	JXA1, TJ, HuN4	China, Southeast Asia	HP-PRRSV-based MLVs	High (homologous)	Partial against some L1 and L2 strains	Limited reproductive protection data; inconsistent outcomes across studies	[77,78,80,81,82,83,84,94]
Lineage 3	QYYZ, SD53	China, Taiwan	None (limited L7 vaccine data)	Not evaluated	Limited; poor neutralization by L5/L8 vaccines	High pathogenicity but scarce vaccine evaluation	[85,86,87,88,89,90,91]
Minor lineages (L2, L4, L6–L7, L9)	NADC-34, MN184A	Regional	L7-based MLV (limited use)	Limited data	Limited data	Sparse experimental evaluation; unclear epidemiological relevance	[91,92]

## 5. Determinants of Protective Immunity

### 5.1. T-Cell-Mediated Immunity in Broad and Heterologous Protection Against PRRSV

T-cell responses play a central role in protection against PRRSV and involve multiple immune components, including T helper (Th) cells, cytotoxic T lymphocytes (CTLs), regulatory T cells (Tregs), TCR-γδ T cells, and IFN-γ–producing T cells [10]. Compared with virus-neutralizing antibody responses, T-cell immunity is generally broader and exhibits greater heterologous reactivity [10,95], although this breadth does not consistently extend across PRRSV species. For example, PRRSV-2 infection induces broadly cross-reactive cell-mediated immunity among PRRSV-2 strains, whereas IFN-γ responses to PRRSV-1 remain minimal, indicating a species-specific limitation [96]. Similarly, among PRRSV-1 strains, the development of cross-reactive IFN-γ-secreting cells is variable across strains [97].

Vaccination with PRRSV-1- or PRRSV-2-based MLVs can induce antigen-experienced CD4^+^, CD8^+^, and CD4^+^CD8^+^ T cells capable of producing IFN-γ and IL-10 upon heterologous restimulation, in some cases at levels comparable to homologous stimulation. These findings support a role for cell-mediated immunity in cross-protection, although the relative contribution of T-cell subsets appears context-dependent. In particular, CD8^+^IFN-γ^+^ responses are often more pronounced than CD4^+^IFN-γ^+^ responses following stimulation with heterologous, highly pathogenic PRRSV-2 strains (lineage 8.7), whereas CD4^+^ T cells—but not CD8^+^ cells—have been implicated as critical for protection against PRRSV-1-associated reproductive disorders [79,98,99].

IFN-γ plays an important antiviral role by inhibiting PRRSV replication and contributing to protection against reproductive disease [98,100]. However, IFN-γ responses are typically delayed and of relatively low magnitude following infection with virulent field strains or MLV vaccination, especially when compared with responses induced by pseudorabies vaccination [101,102]. Memory T cells, predominantly CD3^+^CD4^+^CD8α^+^, account for most IFN-γ-secreting cells and increase following booster vaccination, although overall responses remain modest. Notably, heterologous IFN-γ responses are often undetectable prior to challenge but become enhanced following vaccination and subsequent infection, supporting IFN-γ as a potential correlate of both homologous and heterologous protection [56,103].

The magnitude and quality of IFN-γ responses are strongly influenced by viral strain and pathogenicity. Highly pathogenic PRRSV-1 subtype 3 (Lena) induces stronger IFN-γ responses than low-pathogenic subtype 1 strains [56], and highly pathogenic PRRSV-2 strains generally elicit higher IFN-γ levels than strains such as NADC20, although exceptions (e.g., SD53) have been reported [104,105]. Strain-specific effects are also evident following PBMC restimulation of pigs vaccinated with lineage 5 MLVs, influencing both CD4^+^ and CD8^+^ IFN-γ responses [75].

TCR-γδ T cells contribute to viral control, particularly after peak viremia, suggesting a role in limiting viral persistence and maintaining lymphoid homeostasis [10]. These cells produce pro-inflammatory cytokines, including TNF-α and IFN-γ, and TNF-α-secreting γδ T cells have been associated with protection against PRRSV challenge and reduced transplacental infection, potentially acting at the maternal–fetal interface [98,106]. As with other T-cell responses, γδ T-cell activation appears strain-dependent, particularly following stimulation with diverse PRRSV-2 isolates [75].

Collectively, these findings indicate that T-cell immunity—including IFN-γ-producing CD4^+^, CD8^+^, and TCR-γδ T cells—contributes to both homologous and heterologous protection against PRRSV [10]. However, the magnitude and functional relevance of these responses vary substantially depending on viral strain, pathogenicity, and vaccination context [56,107].

### 5.2. Neutralizing Antibodies in PRRSV: Delayed Kinetics but Critical for Protection

PRRSV infection elicits an atypical humoral immune response characterized by the early dominance of abundant non-neutralizing antibodies (non-NAbs). These early responses are reflected by strong binding antibodies to the viral nucleocapsid (N) protein detected by ELISA, in the absence of corresponding neutralizing activity in serum neutralization assays.

Functional neutralizing antibodies (NAbs) typically arise several weeks after infection [8,9], although a subset of pigs can develop high-titer NAbs as early as 7–28 days post-infection, with marked inter-individual variability even following infection with the same strain [108]. In most cases, NAbs are not detected until 3–4 weeks post-infection [109]. As infection progresses, class switching and affinity maturation result in predominantly IgG neutralizing antibodies targeting the viral envelope glycoproteins GP4, GP5, and the GP5–membrane (M) complex, which mediate receptor binding and membrane fusion [110,111,112,113]. These antibodies can block PRRSV entry into CD163-positive macrophages, thereby preventing productive infection.

In vitro studies demonstrate that sera with high NAb titers efficiently inhibit PRRSV infection in MARC-145 cells and primary macrophages [114,115]. Passive transfer experiments further show that sera or monoclonal antibodies with strong in vitro neutralizing activity can confer complete or near-sterilizing homologous protection in piglets [116,117] in a dose-dependent manner [118]. Substantial reductions in infection have been reported following passive immunization, reaching up to 96% for PRRSV-2 and 87% for PRRSV-1 [116], establishing that under ideal conditions, NAbs alone can prevent productive infection.

However, field studies indicate that measurable neutralizing titers do not reliably predict protection. Vaccinated or convalescent sows with detectable NAbs may still transmit virus vertically, and vaccine-induced NAbs often fail to prevent heterologous infection [119]. Trials with inactivated or autogenous vaccines similarly show that even high NAb titers rarely confer sterilizing protection [51,67]. These discrepancies highlight the extensive genetic and antigenic diversity of PRRSV, which limits antibody breadth and confines protection largely to homologous or closely related strains [120,121].

Notably, protection from challenge infection has frequently been observed in vaccinated pigs in the absence of detectable VN antibodies [38,62,93]. In some cases, monospecific sera failed to neutralize homologous PRRSV-1 strains despite effectively neutralizing heterologous strains [97,122]. Collectively, these observations indicate that both homologous and heterologous protection are context-dependent and cannot be reliably inferred from neutralizing antibody titers alone, underscoring the contribution of other immune components to protection against PRRSV infection [123].

Despite these limitations, broadly neutralizing antibodies (bNAbs) against PRRSV can develop under certain conditions. Passive immunization and serological studies demonstrate that antibodies elicited by infection with one genotype may neutralize heterologous strains [117]. Monoclonal antibodies isolated from hyperimmune sows show cross-reactive neutralization against multiple PRRSV-2 variants, providing direct evidence for naturally arising bNAb-producing B-cell lineages. However, these antibodies generally fail to protect against PRRSV-1 isolates [124]. Similarly, immunization with a chimeric PRRSV-2 containing a consensus ORF2–6 sequence induced broad neutralizing activity in approximately 60% of vaccinated pigs [125], indicating that neutralization breadth is achievable but not universal [126].

Host immune background and exposure history further influence antibody affinity and protective efficacy. Proposed explanations include limited boosting capacity of MLV, lower antigen load or lack of adjuvants compared with inactivated vaccines, and potential immune exhaustion following repeated MLV vaccination, although these mechanisms remain hypothetical [127].

In naïve piglets, maternally derived homologous NAbs can confer near-sterilizing protection by delaying viremia and mitigating clinical disease [128]. However, maternally derived antibodies (MDAs), particularly virus-neutralizing antibodies, can also impair vaccine-induced immune priming by restricting vaccine virus replication. Under certain conditions, such as early vaccination, piglets may partially overcome MDA-mediated interference [129,130].

In addition to systemic neutralizing antibody (VN) responses, mucosal humoral immunity may contribute to protection against PRRSV at primary sites of infection [5]. Anti-PRRSV IgA has been detected in oral fluids, nasal secretions, and bronchoalveolar lavage, with kinetics distinct from serum IgG responses [131]. Moreover, a functional role of mucosal IgA has been demonstrated in vitro, where IgA-rich oral fluid samples reduced PRRSV replication in macrophages [132]. Collectively, these findings highlight the potential of intranasal vaccination to enhance mucosal immune responses, including IgA, which may contribute to improved control of PRRSV at sites of infection.

In summary, PRRSV employs multiple immune evasion strategies that limit the breadth of neutralizing antibody responses (see Section 6.3). Nevertheless, evidence from experimental infection and structural studies indicates that antibodies targeting conserved conformational epitopes represent an important correlate of protection [114,120]. Identifying and exposing such epitopes through rational immunogen design—such as modulation of glycosylation or stabilization of critical glycoprotein complexes—offers a plausible path toward next-generation PRRSV vaccines with broader cross-protection.

## 6. Key Factors Modulating PRRSV Homologous and Heterologous Protection

### 6.1. Impact of Multiple Exposures and Booster Vaccination

Several studies suggest that PRRSV-2 vaccines may induce cross-protection against PRRSV-1 strains more effectively than PRRSV-1 vaccines. This interpretation is supported by observations that sows with multiple PRRSV exposures exhibit high virus-neutralizing (VN) titers against both PRRSV-2 and PRRSV-1 strains [24]. Prolonged antigenic stimulation and/or exposure to a diverse set of infecting strains have been proposed as key drivers of this breadth, consistent with broader principles established in other viral systems in which infection duration contributes to development of broadly active VNAs [133].

Repeated exposure through natural infection or vaccination is likewise associated with higher VNA levels, and broader activity and field data indicate that double vaccination with MLV is often more effective than a single dose, particularly in PRRSV-unstable herds [134,135]. Beyond humoral responses, repeated antigen exposure can strengthen cell-mediated immunity, including IFN-γ-associated responses (see Section 5.1) [136,137]. Sustained antigen exposure is also required for induction of CD4^+^ cytotoxic T lymphocytes, which have been linked to protection against PRRSV infection [98].

However, repeated exposure to antigenically similar strains may blunt immune responsiveness, consistent with a “Hoskins effect” that has been proposed to contribute to suboptimal responses in multi-vaccinated sows [134,135]. Notably, this phenomenon has been reported primarily for MLV vaccination, whereas repeated use of inactivated vaccines has been associated with increased ELISA and VNA titers [127]. Together, these data indicate that multiple exposures—particularly when antigenically diverse—can enhance protection against PRRSV, while overly homologous boosting may have diminishing or negative effects.

### 6.2. Strain-Specific Effect: Pathogenicity, and Immune Induction

PRRSV exhibits pronounced strain-specific effects on the induction of protective immunity. Some strains induce broad neutralizing responses, whereas others fail to elicit robust VNAs even against homologous viruses [138]. Innate and adaptive immune polarization also varies by strain, including differences in IFN-α induction [139] and Th1-biased responses [56]. These outcomes are likely driven by differences in viral replication kinetics, cellular tropism, and tissue distribution in vivo [56,140].

HP-PRRSV strains exemplify the relationship between viral fitness and immune priming. These strains have been shown to replicate to higher levels both in vitro and in vivo, and MLVs derived from these strains can confer partial heterologous protection against divergent PRRSV-2 challenges such as NADC-20 and NADC-30 [80,141]. Vaccination with HP-PRRSV MLVs is associated with enhanced cell-mediated responses, including IFN-γ production [107]. Similarly, highly pathogenic PRRSV-1 subtype 3 (Lena) induces strong Th1 response [142], further supporting a link between replication capacity, pathogenicity, and the magnitude and breadth of immune responses.

Collectively, these findings suggest that viral strain biology—including replication efficiency and tissue tropism—plays a central role in shaping immune priming and may help explain the generally greater heterologous and cross-protective potential observed for some PRRSV-2 strains relative to PRRSV-1.

### 6.3. Structural Determinants, Complement, and Rational Immunogen Design

Structural features of PRRSV envelope proteins contribute to delayed (see Section 5.2) and strain-specific neutralization. The GP5 ectodomain contains a decoy epitope adjacent to the principal neutralizing site, diverting early responses toward non-protective regions [143]. In addition, GP3/GP5 glycosylation can create a functional “glycan shield,” limiting access to conserved neutralizing epitopes; glycan removal or modification increases neutralization sensitivity and can enhance homologous NAb induction, including for inactivated vaccines [144,145,146]. Mapping studies implicate conformational epitopes within GP5–M and GP2–GP3–GP4 complexes as key bNAb targets, shaped by folding and glycan positioning [110,147,148]. Complement can markedly increase measured VN titers, indicating that complement availability can modulate neutralization readouts and potentially protection [149,150]. These concepts have motivated immunogen engineering strategies (e.g., chimeric/mosaic designs) to broaden humoral and cellular immunity [151,152,153]. Evidence for consistent cross-strain broadening by glycan manipulation remains limited, but analogous approaches in other viral systems support the plausibility of exposing conserved epitopes to expand breadth [154,155].

### 6.4. Vaccination Context and Host Factors

Vaccination can shape viral population dynamics; vaccinated/challenged pigs may develop more heterogeneous viral populations and greater divergence than non-vaccinated controls [156]. Host variability also contributes, as only a subset of animals mount broadly cross-reactive neutralizing responses [157]. Because most vaccine-efficacy studies use PRRSV-naïve pigs, protection should also be evaluated in endemic settings where preexisting immunity interacts with vaccination. In these contexts, vaccination alone may yield limited VNA responses, while post-vaccination challenge can drive substantial VN boosting [25,121]. Autogenous inactivated vaccines may broaden VN responses against local strains and reduce viremia while boosting existing immunity [65,66,68], supporting targeted booster strategies for herd-level control.

### 6.5. Preexisting Immunity to PRRSV: Potential Drawbacks

Although this review primarily focuses on cross-protection, the potential drawbacks of preexisting immunity also warrant consideration. In particular, two broad mechanisms may contribute to suboptimal or adverse outcomes following heterologous challenge: antibody-dependent effects, including antibody-dependent enhancement (ADE), and broader immune-mediated mechanisms involving dysregulated or poorly protective cellular immune responses. ADE has been proposed as a theoretical risk when vaccination or prior infection fails to generate robust neutralizing immunity and instead produces predominantly non-neutralizing or sub-neutralizing antibody responses.

In vitro studies have demonstrated that sub-neutralizing concentrations of PRRSV-specific antibodies can enhance viral replication in porcine alveolar macrophages through Fc receptor-mediated uptake, accompanied by reduced production of type I interferons and tumor necrosis factor-α [158,159,160]. These findings provide mechanistic support for antibody-facilitated infection under defined experimental conditions. However, the in vivo relevance of ADE in PRRSV infection remains unclear and controversial. Several experimental infection and vaccine-challenge studies have reported instances in which vaccinated or previously infected animals developed disease severity comparable to, or in some cases exceeding, that of naïve controls following heterologous challenge [75,161,162]. In some instances, these outcomes were associated with high levels of binding antibodies, including nucleocapsid-specific responses, in the absence of detectable or robust neutralizing activity. Notably, such effects appear to vary across PRRSV strains rather than be strictly correlated with genetic similarity, as vaccination may confer protection against one strain while having minimal or even adverse effects against another [75].

At the same time, other studies have failed to confirm a direct role for either neutralizing or non-neutralizing antibodies in enhanced disease, instead suggesting that observed outcomes may reflect strain-specific virulence differences or other immunological factors unrelated to classical ADE mechanisms [61]. Notably, in vitro evidence of enhanced replication does not necessarily translate into increased disease severity in vivo. For example, one study demonstrated increased replication of a heterologous PRRSV strain following incubation with sera from MLV-vaccinated animals, yet it did not observe corresponding evidence supporting antibody-mediated effects on disease severity in vivo [161].

Cytokine responses play a central role in PRRSV infection, including T-cell-mediated immunity (see Section 5.1), strain-specific differences in type I interferon regulation [162] and vaccine-specific cytokine profiles such as IL-10 responses [107]. These factors collectively shape immune outcomes and suggest that mechanisms beyond non-neutralizing antibodies may contribute to suboptimal or dysregulated responses following heterologous challenge.

## 7. Summary and Future Perspectives

Despite decades of effort, current PRRS vaccines provide inconsistent heterologous protection, underscoring the need for next-generation strategies that move beyond strain-matched approaches. Achieving broad, durable protection against PRRSV will require a deeper understanding of the immunological and viral determinants that govern cross-protection, including neutralizing antibody breadth, T-cell-mediated immunity, antigenic distance, and viral immune evasion mechanisms. Importantly, protection does not follow a strictly hierarchical pattern: although homologous immunity is generally more robust, it is not universally predictive of optimal outcomes, and heterologous protection can at times exceed expectations.

Future vaccine development should prioritize mechanistic correlates of protection rather than genetic relatedness alone. While serum-neutralizing antibody (VN) responses remain a central metric of vaccine efficacy, accumulating evidence suggests that they may not fully capture protective immunity, particularly at primary sites of infection. In this context, mucosal immune responses—including local IgA and tissue-associated cellular immunity—may play an important complementary role in limiting early viral replication and transmission. Integrating systemic and mucosal immunity into a unified framework of protection may therefore provide a more accurate basis for evaluating vaccine performance and guiding vaccine design.

One rational direction is to evaluate sequential immunization, in which an antigenically enriched PRRSV-2 strain is used for priming, followed by a boost with the strain of highest regional or clinical relevance. Because PRRSV immunity is often strain-specific, selection of the priming strain should emphasize immunological performance—such as the capacity to induce broader neutralizing antibody responses and cross-reactive T-cell immunity—rather than sequence homology alone. Incorporating strategies that enhance mucosal immunity, including alternative routes of administration or adjuvant design, may further improve protective outcomes. Subsequent boosting with a field-relevant strain may then enhance protective magnitude while preserving immune breadth, potentially resulting in more robust and durable protection under field conditions. Systematic comparative evaluation of such strategies will be essential to determine whether breadth-focused priming followed by strain-relevant boosting improves heterologous protection relative to conventional strain-matched approaches.

In addition, several emerging approaches offer promising avenues for next-generation PRRSV vaccine design. Structure-guided antigen design may enable the identification and stabilization of conserved neutralizing epitopes, facilitating the induction of broader antibody responses across divergent strains. Similarly, T-cell-focused vaccine strategies, including the identification and incorporation of conserved CD4^+^ and CD8^+^ T-cell epitopes, may enhance cross-reactive cellular immunity and improve protection against heterologous challenge. Advances in reverse genetics and synthetic biology also provide opportunities to rationally engineer vaccine strains with improved immunogenic profiles, reduced immune evasion capacity, and enhanced safety.

Finally, integrating antigen design with improved delivery platforms—such as viral vectors, nanoparticle-based vaccines, or multivalent formulations—may further enhance immune breadth and durability. Collectively, these approaches highlight a shift from empirically derived vaccines toward rationally designed immunogens and vaccination strategies tailored to overcome the antigenic diversity and immune modulation characteristic of PRRSV.

## Figures and Tables

**Table 1:** Reference PRRSV ORF5 Strains Used for Phylogenetic Analysis.

Subtype/Lineage	Specific Vaccine Available	Sublineage	Representative Strain	GenBank Accession
PRRSV-1				
Subtype 1-1	yes	na	Lelystad (LV)	M96262
			Spanish R1	OM893828
			AUT15-33	MT000052
			PR40/2014	MF346695
			MLV-DV	KJ127878.1
			Unistrain-VP-046-bis	MK134483.1
			94881	KT988004.1
Subtype 1-2	no		WestSib13	KX668221.1
Subtype 1-3	no		Lena	JF802085
			SU1-Bel	KP889243
PRRSV-2				
Lineage 1	yes	1A	NC174	PP171544
		1C	NADC30	JN654459.1
		1C.5	USA/MN/01775GA/2021	OR634972.1
			RFLP 1-4-4 variant	MW887655
			UIL21-0712	PQ810800
		L1D	Prevacent^®^ PRRS	KU131568
		L1A	NADC34	MF326985
		L1H	USA/81793-6/2019	OR634975
Lineage 3	no	3.5	QYYZ	JQ308798
		3.5	GM2	JN662424.1
Lineage 5	yes	5A	VR-2332	EF536003.1
			RespPRRS MLV	AF066183
		5B	NADC-8	AF396833
Lineage 7	yes	na	Prime Pac^®^ PRRS RR	DQ779791
Lineage 8	yes	8.7	JXA1	EF112445
		8.7	TJ	EU860248
		8.7	TP P90	GU232737
		8C	Fostera^®^ PRRS	KP300938
		8A	Ingelvac PRRS^®^ ATP	DQ988080
Lineage 9	no	na	NADC-20	JX069953

**Table 2:** Experimental evidence for cross-species protection between PRRSV-1 and PRRSV-2.

Primary Immunization/Exposure	Challenge Virus	Experimental Model	Protection Endpoints Assessed	Primary Protection Domain	Main Outcome	Key References
PRRSV-2 MLV	PRRSV-1	Growing pigs	Clinical signs, viremia, lung lesions	Clinical > Virological	Partial protection: reduced clinical severity and viremia	[27,28]
PRRSV-2 MLV	PRRSV-1	Pregnant sows/gilts	Reproductive performance, viremia	Reproductive (partial)	Partial protection: improved reproductive outcomes but incomplete virological protection	[29]
PRRSV-2 MLV	PRRSV-1	Boars	Virus shedding in semen	None	Limited protection: reduced PRRSV-2 shedding only	[26]
PRRSV-2 MLV	PRRSV-1	Nursery pigs	Clinical disease, viremia	Variable/endpoint-dependent	Inconsistent: protection varied by strain and endpoint	[28]
PRRSV-1 MLV	PRRSV-2	Growing pigs	Clinical disease, viremia, shedding	None	No or minimal protection	[35,36]
PRRSV-1 MLV	PRRSV-2	Boars	Semen shedding	None	No protection against PRRSV-2 shedding	[36]
PRRSV-1 MLV	PRRSV-2	Nursery pigs	Clinical signs	Clinical only	Partial clinical protection (no virological protection)	[38]
PRRSV-1 MLV followed by PRRSV-2 MLV (consecutive)	PRRSV-1 + PRRSV-2	Nursery pigs	Clinical signs, viremia	Bidirectional (partial)	Improved bidirectional protection compared with single-species vaccination	[30]
Natural PRRSV-2 infection	PRRSV-1	In vitro (MARC-145 cells)	Viral replication	Virological (in vitro)	Inhibition of PRRSV-1 replication after PRRSV-2 pre-infection	
Natural PRRSV-1 infection	PRRSV-2	In vitro (MARC-145 cells)	Viral replication	None (in vitro)	No inhibition of PRRSV-2 replication	
PRRSV-2 MLV	PRRSV-1	Late-gestation sows	Reproductive outcomes	None	No protection	[25]
PRRSV-1 or PRRSV-2 MLV	PRRSV-1 + PRRSV-2 dual challenge	Growing pigs	Clinical signs, viremia	Asymmetric	Limited and asymmetric protection, favoring PRRSV-2 priming	[37]

## Data Availability

No new data were created or analyzed in this study.
